# Quantifying holistic capacity response and healthcare resilience in tackling COVID-19: Assessment of country capacity by MCDM

**DOI:** 10.1371/journal.pone.0294625

**Published:** 2024-04-05

**Authors:** Dilber Uzun Ozsahin, Nuhu Abdulhaqq Isa, Berna Uzun, Ilker Ozsahin

**Affiliations:** 1 Medical Diagnostic Imaging Department, College of Health Sciences, University of Sharjah, Sharjah, United Arab Emirates; 2 Operational Research Center in Healthcare, Near East University, Nicosia/TRNC, Mersin, Turkey; 3 Department of Biomedical Engineering, Faculty of Engineering, Near East University, Nicosia/TRNC, Mersin, Turkey; 4 Department of Biomedical Technology, Nasarawa State College of Health Science and Technology, Keffi, Nasarawa State, Nigeria; 5 Department of Statistics, Carlos III University of Madrid, Madrid, Spain; 6 Brain Health Imaging Institute, Department of Radiology, Weill Cornell Medicine, New York, New York, United States of America; Public Library of Science, UNITED KINGDOM

## Abstract

The resilience of a country during the COVID-19 pandemic was determined based in whether it was holistically prepared and responsive. This resilience can only be identified through systematic data collection and analysis. Historical evidence-based response indicators have been proven to mitigate pandemics like COVID-19. However, most databases are outdated, requiring updating, derivation, and explicit interpretation to gain insight into the impact of COVID-19. Outdated databases do not show a country’s true preparedness and response capacity, therefore, it undermines pandemic threat. This study uses up-to-date evidence-based pandemic indictors to run a cross-country comparative analysis of COVID-19 preparedness, response capacity, and healthcare resilience. PROMETHEE—a multicriteria decision making (MCDM) technique—is used to quantify the strengths (positive) and weaknesses (negative) of each country’s COVID-19 responses, with full ranking (net) from best to least responsive. From 22 countries, South Korea obtained the highest net outranking value of 0.1945, indicating that it was the most resilient, while Mexico had the lowest (-0.1428). Although countries were underprepared, there was a robust response to the pandemic, especially in developing countries. This study demonstrates the performance and response capacity of 22 key countries to resist COVID-19, from which other countries can compare their statutory capacity ranking in order to learn/adopt the evidence-based responses of better performing countries to improve their resilience.

## Introduction

Historically, humans have always been vulnerable to outbreaks, epidemics, and pandemics. Pandemics are regarded as the most significant of global health emergencies. COVID-19 started as an outbreak in Wuhan, then became an epidemic in China, and spread as far as Europe. On March 2020, the virus was declared a pandemic of international concern [1], having met the epidemiological criteria of > 100,000 infections in at least 100 countries [2]. COVID-19 was characterized by several initial index cases, then an exponential sporadic spread [3], bringing unprecedented global changes. The amendment enacted by International Health Regulations by the WHO [4] requires all countries to provide vital resources to prepare for outbreaks, epidemics, and pandemics [1]; however, there is a low level of commitment to the act. The emergence of the COVID-19 pandemic emphasized this lack of commitment through the resulting impacts felt in every country. Perhaps more disturbingly, another outbreak, epidemic, or pandemic could emerge at any time from cases of Orthohantavirus (Hantavirus), zika, dengue virus, and Lassa fever.

COVID-19 spread rapidly resulting in uncertainty in terms of its nature, pathology, prognosis, and measures with which it could be tackled. The implementation of COVID-19 measures was directly related to incidence and mortality rates [1]. There was no uniform measure for mitigating COVID-19 [5, 6]; hence, countries were required to customize measures according to their existing healthcare systems and resources. COVID-19 measures are pharmacological and non-pharmacological [7], where the former relates to medical intervention, while the latter relates to public health mitigation measures like lockdowns and movement restrictions [8]. According to Fernandes [9], for any infectious threats, healthcare systems constitute the frontline defense, which if not resilient, will ultimately collapse due to overwhelming resource shortages against rising cases, a reality that was observed during the COVID-19 pandemic [10]. Developed countries’ hospital systems were significantly overwhelmed [11], while developing countries were chronically underprepared for COVID-19 [12]. The high incidence, mortality, and overwhelmed healthcare systems in developed countries revealed the vulnerability of global health systems. Thus, previous knowledge of countries’ health system capacity to cope with major public health challenge was incomplete or incorrect, raising important questions on how countries’ preparedness is measured [13].

Countries claimed to demonstrate the best performance should be considered models for COVID-19 response. Independent reports and researches have also comparatively analyzed the global COVID-19 response, providing insights on response models worthy of emulation. For example, Hale et al. [14] developed the Oxford Covid-19 Government Response Tracker (OXCGRT) to keep track of cross-country responses. It consists of a government response stringency index, which comprises several indicators measured on an ordinal scale—the higher the score the stricter a government’s response is. The European Union’s COVID-19 health systems response monitor (HSRM) was developed for collecting, organizing and comparatively analyzing countries’ response/performance [15]. In the UAE, Moonesar et al. [16] improved on the OXCGRT approach, using 17 indicators, to evaluate 8 countries from different geographical settings with distinct socio-economic characteristics. Deep Knowledge [17] used public data to determine the countries around the world considered the safest, riskiest and with the most efficient treatment with regard to COVID-19. A big data analytical framework with COVID-19 indicators compared 40 countries. Israel was determined to be the safest and Slovenia the least safe. For COVID-19 risk level and treatment efficiency, 150 countries were analyzed using 76 indicators. Italy was ranked the riskiest, followed by the USA, UK, Spain, France, Germany, China, and South Korea. After reviewing these studies, we found that the data used were very outdated, with some as old as 2005, and were therefore incapable of providing a clear picture of the COVID-19 reality. Therefore, databases should regularly be updated, especially when researchers require them to decode the mysteries surrounding multi-faceted pandemics. Bill Gates in 2015 recommended disease surveillance to ensure that appropriate databases are readily available [18].

Our study collected updated evidence-based COVID-19 indicators to derive, quantify, and provide explicit interpretations of the responses/performance of 22 countries. A cross-country comparative analysis was run to quantitatively rank countries’ responses and healthcare resilience. Fuzzy-PROMETHEE—an MCDM technique—provides the full ranking (showing the strengths and weakness) of alternatives, from the best to least performing alternatives using multiple criteria [19]. Fuzzy logic handles imprecise/uncertain, deficient, and existing data [20]. It also allows for imprecise input and few rules to encompass problems with great complexity [21]. For this reason, the implementation of fuzzy logic in our study allowed for any imprecise/uncertain, or even deficient data, to be handled, which was useful considering the uncertain nature of COVID-19. PROMETHEE is an outranking method with several iterations that ranks alternatives according to multiple-criteria. Fuzzy-PROMETHEE deals with incomplete, vague, and uncertain data. By exploiting the excellent properties of fuzzy and PROMETHEE, a full ranking of alternatives can be achieved even with uncertain or incomplete data. Additionally, the reason for choosing this method for our study is because of its effectiveness and user-friendliness. It gives the researcher room to achieve certain goals without complicating the algorithmic process. In this study, countries were the alternatives, while the COVID-19 response indicators were the criteria.

There are several analytical MCDM techniques available for decision-makers, each of which has its own set of advantages and disadvantages. PROMETHEE stands out by offering a comprehensive analysis that involves pairwise comparisons between options and criteria. By employing different preference functions, PROMETHEE enables decision-makers to obtain more sensitive ranking results, effectively determining the superiority of one alternative over others. Additionally, PROMETHEE considers the interactions between alternatives and accurately captures the preference indices of alternatives for each criterion, providing a thorough understanding of the choice space. Furthermore, PROMETHEE offers visualization of the advantages and disadvantages associated with each alternative, enhancing transparency compared to other options. It is also suitable for both qualitative and quantitative data analysis. Moreover, PROMETHEE facilitates sensitivity analysis, enabling decision-makers to explore the robustness of the findings. This allows them to assess the impact of changing criteria weights or using alternative evaluations, providing valuable insights into the decision-making process. The data we collected to determine the effectiveness of the countries’ responses to COVID-19 was in a form that fits well with PROMETHEE. Considering the numerous successful applications of the PROMETHEE model and its associated advantages, we chose this approach for our analysis.

Fuzzy and PROMETHEE have both been applied in healthcare in the past decade [22]. Sayan et al. [23] solved uncertainties in COVID-19 diagnostic approaches by using fuzzy-PROMETHEE. Alsalem et al. [24] reviewed literature on the role of multi-attribute decision making (MADM) in tackling the COVID-19 pandemic. Articles were collected from major databases with a focus on the application of MADM in medical, social, economic, and technological fields. In another review, Alsalem et al. [25] identified the lack of decision mechanisms during the COVID-19 outbreak. The theoretical review emphasized the potential of multi-criteria decision making (MCDM) techniques/theories significant in the fight against COVID-19. The study recommended the Fuzzy Delphi method, Fuzzy weighted Zero Inconsistency, and Fuzzy Decision by Opinion Score method, as future directions in COVID-19 studies. Zizovic et al. [26] utilized a two-phase model (with separate multiple-criteria model in each phase) to address some of the challenges faced during the pandemic. The study evaluated and selected nurses for COVID-19 shift hospitals in an attempt to identify those that completely satisfied the requirements for working in such hospitals and those that required additional training to meet the requirements. In an attempt to identify the most vulnerable aspect of high-income countries in terms of COVID-19, Tuzcu and Türkoğlu [27] used a novel MCDM technique called the Proximity Indexed Value (PIV) method. The main criteria used were politics, demography, capacity, and Covid-19 indicators. The findings showed that countries with less equitable healthcare systems and with more vaccine hesitancy were more vulnerable to COVID-19. Katanić and Damjanović [28] found significant cross-country differences in people’s mobility during the COVID-19 pandemic due to differences in government policies restricting people’s movement in the past two years. The Fuzzy decision by opinion score method (FDOSM) and fuzzy-weighted zero inconsistency (FWZIC) were used to solve several issues related to COVID-19, including the prioritization of COVID-19 patients for Mesenchymal stem cell treatment [29] and prioritizing COVID-19 vaccine dose recipients [30].

Countries need to prepare and expand their health response capacity using indicators such as hospital beds, doctors, nurses, and the GHS index [31]. Other indicators include human resources, medical supplies, and emergency capacity [32]. When facing an unexpected epidemic, governments are expected to strengthen their healthcare workforce (HCWs), operational efficiency, industrial capacity to produce medical resources/facilities, and citizen solidarity. These indicators determine a country’s capacity to minimize the burden of epidemics [33]. One of the greatest risks to the healthcare system is the potentially high rate of COVID-19 infections among HCWs, which reduces the capacity for a local and/or regional response to the pandemic [34].

In this study, we address the need for a holistic assessment of countries’ preparedness, response capacity, and healthcare resilience in tackling the COVID-19 pandemic. We recognize that traditional databases often lack up-to-date information and fail to provide a comprehensive understanding of a country’s true preparedness and response capacity. Therefore, we utilize evidence-based pandemic indicators and employ the fuzzy PROMETHEE technique as a MCDM approach to quantitatively evaluate the strengths and weaknesses of 22 countries’ COVID-19 responses. Overall, the main contribution of this study is the application of fuzzy PROMETHEE to assess and quantify the holistic capacity response and healthcare resilience of countries in tackling COVID-19, providing valuable insights for policymakers and health authorities to strengthen their pandemic response strategies.

## Materials and methods

To analyze country responses, the systematic collection of accurate, consistent, and complete data is required [14, 31]. Publicly available data were sourced from the WHO, CDC, NCDC, Hopkin hospital, 2019 GH index, Epidemic Preparedness Index [35], health ministries, private organizations, and news outlets etc. As a result of the COVID-19 dynamics, data were only collected for specific periods for all countries. Data were derived, transformed, and quantified, for 22 countries including Brazil, China, Egypt, France, Germany, India, Israel, Italy, Mexico, New Zealand, Nigeria, Qatar, Russia, Saudi Arabia, South Africa, South Korea, Spain, Sudan, Turkey, UAE, UK, and USA. Data were run using the Fuzzy-PROMETHEE method. This method supports the linguistic nature of COVID-19 data since the fuzzy scale is capable of working with data within ranges, vagueness, and qualitative [23]. The ranking system is based on competing alternatives coupled with quantified data derived into 38 criteria, as shown in Eq 4.2. These criteria are divided into 4, scenarios as shown in Table 1.

**Table 1:** Ranking COVID-19 response indicators (criteria) sorted in to four scenarios.

Scenario 1	Scenario 2	Scenario 3	Scenario 4
Resources	Medical response	Healthcare workers (HCWs)	COVID-19 status
Test Laboratory	Test/P	Doctors/1k	Total cases (TC)
Test Lab/P	Dependence	Transparency	Total cases/ P (TC/P)
ICU beds	Innovations	Nursing/1k	Active cases (AC)
ICU beds/P	Vaccines	Infected HCWs	Active cases/TC (AC/TC)
ICU beds/TC	Lockdown	Dead HCWs	Total deaths (TD)
Ventilators	Emergency fund	HCWs’ infection rate %	Total deaths/TC (TD/TC)
Ventilators/P	Emergency fund/P	HCWs’ mortality rate %	Total recovered (TR)
Ventilators/TC	Emergency fund/TC	Biomedical engineers/1k (BMEs/1k)	Total recovered/TC (TR /TC)
Hospital beds	Vaccine doses		Transparency
Hospital beds/1k	Vaccine doses/P		
Health system (HSY)	Transparency		
Health insurance (HI)			
Transparency	*P is population		

### Fuzzification of data

Data were defined using a triangular-fuzzy linguistic scale to obtain the importance weight of each criterion. Weighting was significantly influenced by the literature review and expert specifications. Weighted data were transformed into triangular-fuzzy numbers for fuzzification, as provided in [36]. The Yager index—a rational technique—was applied to defuzzify fuzzy sets based on the calculation of their center. At this point, the dataset is ready for simulation in PROMETHEE. Detailed datasets, sources, list of COVID-19 indicators, triangular and linguistic scale preference, and weights of data used to support the findings of this study have been deposited in the 4TU.ResearchData repository [36], under CC0 license.

In this study, a triangular fuzzy scale was used to numerically determine linguistic data for imprecise parameters. Zadeh [37] defined fuzzy sets for defining linguistic or vague data mathematically based on membership degrees. Triangular, rectangular, and Gaussian fuzzy sets are the most commonly used fuzzification processes. Based on our experience with crisp data, once the normalization process was applied for normal data, we still obtained the same defuzzification values for the proposed triangular fuzzy sets. Even after using different fuzzy sets, we still obtained the same defuzzified point for the proposed linguistic scale, and therefore, the results would not change significantly. In a fuzzy environment, there are some limitations. The process of defining and weighing vague or imprecise data as a fuzzy number or set is at the discretion of the decision-maker(s), creating room for possible bias, which is a limitation for any method. In practice, since it is difficult to obtain crisp data in most systems, fuzzy logic pre-processing allows data scientists to analyze systems with imprecise information, and triangular fuzzy sets are one method of fuzzifying the imprecise environment.

### Process of fuzzy-PROMETHEE

Gaussian preference function was chosen to determine the net ranking results, as it is resistant to minute and inconsequential deviations in input values [38]. As stipulated by Brans et al. [39, 40] and Geldermann et al. [41], the five main processes of PROMETHEE analysis include;

1. Define the preference function (Gaussian) p_j_ for each criterion j for all criteria.
2. Each criterion should be weighted according to importance $w_{T}\;=\;(w_{1},\;w_{2},\ldots ,w_{k})$,
$$\sum_{i=1}^{k}w_{k}=1,\;where\;(T=1,2,\ldots ,k\;and\;k\;is\;the\;number\;of\;criteria).$$ (4.1)
3. The outranking relation for each a_t_, a_t′_ ∈ A must be deifined using
$$\pi (a_{t},a_{t^{\prime }})=\sum_{k=1}^{K}w_{k}.[p_{k}(f_{k}(a_{t})-f_{k}(a_{t^{\prime }}))],\;AXA\to [0,1]$$ (4.2)
  Where π (a,b) is the preference index- the preference degree of a_t_ to a_t′_ in the study.
4. Eqs (4.3) and (4.4) are used to determine the leaving (positive) and entering (negative) outranking flows respectively for each a_t_
$$\Phi^{+}(a_{t})=\frac{1}{n-1}\sum_{\begin{array}{c} t^{\prime }=1\\ t^{\prime }\neq t \end{array}}^{n}\pi (a_{t},a_{t^{\prime }})\;(n=number\;of\;alternatives)$$ (4.3)
$$\Phi^{-}(a_{t})=\frac{1}{n-1}\sum_{\begin{array}{c} t^{\prime }=1\\ t^{\prime }\neq t \end{array}}^{n}\pi ({a_{t^{\prime }},a}_{t})\;(n=number\;of\;alternatives)$$ (4.4)
  The comparative analysis for each alternative among the alternatives is n-1. Furthermore, Φ^+^(a_t_) which is the positive outranking flow, signifies the strength of alternative a_t_∈A, while Φ^−^(a_t_), the negative outranking flow, signifies the weakness.
5. The principle below should be employed to find the partial preorder on alternative A. If Eq 4.5 is met, then a_t_ is more preferable to a_t′_ (a_t_Pa_t′_) in case of PROMETHEE I
6.
$$\left\{ \begin{array}{c} \Phi^{+}(a_{t})>\Phi^{+}(a_{t^{\prime }})\;\mathrm{and}\;\Phi^{-}(a_{t})<\Phi^{-}(a_{t^{\prime }})\\ \Phi^{+}(a_{t}){>\Phi }^{+}(a_{t^{\prime }})\;\mathrm{and}\;\Phi^{-}(a_{t})=\Phi^{-}(a_{t^{\prime }})\\ \Phi^{+}(a_{t})=\Phi^{+}(a_{t^{\prime }})\;\mathrm{and}\;\Phi^{-}(a_{t}){<\Phi }^{-}(a_{t,}a_{t^{\prime }})\; \end{array}\right.$$ (4.5)
  When two alternatives a_t_ and a_t′_ have the same leaving and entering flows, a_t_ is indifferent to a_t′_ (a_t_Ia_t′_):
$$(a_{t}Ia_{t^{\prime }})if:\Phi^{+}(a_{t})=\Phi^{+}(a_{t^{\prime }})\;and\;\Phi^{-}(a_{t}){=\Phi }^{-}(a_{t^{\prime }}).$$
  a_t_ is incomparable to a_t′_ (a_t_Ra_t′_) if;
$$\left\{ \begin{array}{l} \Phi^{+}(a_{t})>\Phi^{+}(a_{t^{\prime }})\;\mathrm{and}\;\Phi^{-}(a_{t})>\Phi^{-}(a_{t^{\prime }})\\ \Phi^{+}(a_{t})<\Phi^{+}(a_{t^{\prime }})\;\mathrm{and}\;\Phi^{-}(a_{t})<\Phi^{-}(a_{t^{\prime }}) \end{array}\right.$$ (4.6)
7. Using Eq (4.7), the net outranking flow is obtained across the criteria
$$\Phi^{net}(a_{t})=\Phi^{+}(a_{t})-\Phi^{-}(a_{t})$$ (4.7)
  By PROMETHEE II, Eqs (4.8) and (4.9) can provide complete preorder from net flow.
$$a_{t}\;is\;preferred\;to\;a_{t^{\prime }}\;(a_{t}Pa_{t^{\prime }})\;if\;\Phi^{net}(a_{t})>\Phi^{net}(a_{t^{\prime }})$$ (4.8)
$$a_{t}\;is\;indifferent\;to\;a_{t^{\prime }}\;(a_{t}Ia_{t^{\prime }})\;if\;\Phi^{net}(a_{t})=\Phi^{net}(a_{t^{\prime }}).$$ (4.9)
  The higher the Φ^net^(a_t_) value, the better the competitive rank.

## Result and discussion

PROMETHEE analysis provides the ranking of COVID-19 responses in 22 selected countries. The ranking is based on competing alternatives with quantitative data derived from 38 criteria. Table 2 shows the positive, net, and negative outranking values across all countries. Positive values indicate each countries’ COVID-19 response strengths, and should be as high as possible, while negative values signify cumulative weakness, and should be low as possible. The net flow is the distance between the positive and negative flow, providing full ranking results—the country with the highest net value has the best COVID-19 performance and vice versa in 4 scenarios: preparedness, COVID-19 status, response, and HCWs’ status. Table 2 shows the ranking of the countries’ COVID-19 preparedness.

**Table 2:** Ranking results of the countries based on their healthcare resources.

Scenario 1 Resources (Preparedness)									
Rank	Country	Net	Phi+	Phi-	Rank	Country	Net	Phi+	Phi-
**1**	USA	0.2208	0.2803	0.0595	**12**	New Zealand	0.0221	0.2231	0.2011
**2**	Germany	0.2154	0.2885	0.0731	**13**	South Africa	-0.0073	0.1594	0.1667
**3**	South Korea	0.2091	0.2863	0.0771	**14**	Saudi Arabia	-0.0180	0.1479	0.1659
**4**	UK	0.1217	0.2262	0.1045	**15**	India	-0.0272	0.1538	0.1810
**5**	France	0.1213	0.2291	0.1079	**16**	Israel	-0.0404	0.1338	0.1742
**6**	Turkey	0.1069	0.2100	0.1030	**17**	Mexico	-0.1099	0.1117	0.2216
**7**	China	0.0807	0.2220	0.1413	**18**	Egypt	-0.1719	0.0896	0.2615
**8**	Italy	0.0782	0.2043	0.1261	**19**	UAE	-0.2026	0.0612	0.2638
**9**	Spain	0.0749	0.1983	0.1234	**20**	Qatar	-0.2117	0.0570	0.2687
**10**	Brazil	0.0639	0.2032	0.1393	**21**	Nigeria	-0.2611	0.0408	0.3018
**11**	Russia	0.0621	0.2106	0.1485	**22**	Sudan	-0.3270	0.0130	0.3400

USA had the highest net value making it most prepared country with necessary resources to holistically tackle COVID-19. Germany and South Korea were proximally ranked 2^nd^ and 3^rd^. respectively. These are the top three most prepared countries with sufficient resources to detect, stop, and prevent pandemic threats. Compared to the Global Health Security (GHS) index [31], USA was most prepared, then South Korea, and Germany. The UK was ranked 2^nd^ after the US in the GHS index ranking; however, in our study, the UK ranked 4^th^ below South Korea and Germany. France and Turkey are among the top six most prepared countries. China, Italy, Spain, and Brazil are among top ten most prepared countries, followed by Russia, New Zealand, South Africa, Saudi Arabia, India, Israel, Mexico, Egypt, UAE, and Qatar, accordingly. No African countries are among top 10 most prepared, and only South Africa and Egypt were in the top 20 ranked 13^th^ and 18^th^. Nigeria and Sudan ranked 21^th^ and 22^th^ respectively; these two subs–Saharan African countries were the least prepared. Comparatively, both our study and the 2019 GHS index ranked USA as the most prepared country for infectious diseases like COVID-19; however, our ranking of country preparedness differs from the GHS index ranking, potentially due to the extensive use of outdated data in the GHS analysis. The results for Scenario 1 indicate the possibility of inaccuracy in previous measurements of country preparedness, health capacity and resources, as raised by LaFortune [13].

### Scenario 2 Medical response

The ranking for scenario is shown in Table 3.

**Table 3:** Result of scenario 2 (medical response) ranking criteria.

Scenario 2 Medical response									
Rank	Country	Net	Phi+	Phi-	Rank	Country	Net	Phi+	Phi-
**1**	Germany	0..2237	0.2980	0.0743	**12**	UAE	-0.0071	0.0908	0.0979
**2**	China	0..1872	0.2822	0.0949	**13**	Saudi Arabia	-0.0129	0.1409	0.1538
**3**	Spain	0..1357	0.2172	0.0815	**14**	Mexico	-0.0439	0.1361	0.1800
**4**	UK	0..1062	0.2020	0.0958	**15**	India	-0.0547	0.1171	0.1718
**5**	South Korea	0..0982	0.2144	0.1162	**16**	France	-0.0719	0.1046	0.1765
**6**	Brazil	0..0888	0.2238	0.1350	**17**	New Zealand	-0.0719	0.1332	0.2051
**7**	Italy	0..0723	0.2025	0.1303	**18**	Russia	-0.0789	0.1429	0.2218
**8**	USA	0..0480	0.1789	0.1309	**19**	Sudan	-0.1128	0.1064	0.2192
**9**	South Africa	0..0086	0.1702	0.1616	**20**	Israel	-0.1610	0.0773	0.2383
**10**	Qatar	0..0047	0.1008	0.0960	**21**	Nigeria	-0.1657	0.0745	0.2402
**11**	Turkey	0..0042	0.1728	0.1686	**22**	Egypt	-0.1967	0.0585	0.2552

Germany is the most responsive to COVID-19, with the highest positive and lowest negative value. Medical response signifies the effective use of resources to respond to the pandemic. While the USA is most prepared country in Scenario 1, its response was ranked 8th. This lack of translation of preparedness to COVID-19 responsiveness is a matter of debate in academia. The top ten most responsive countries include China, Spain, UK, South Korea, Brazil, Italy, USA, South Africa, and Qatar. This result reveals a rigorous competitiveness; while a country may perform better than another country in one indicator, it is inferior in another indicator. For example, China administered the most vaccine/P, however, had the highest dosage/P. The ranking continues with Turkey, UAE, Saudi Arabia, Mexico, India, France, New Zealand, Russia, Sudan, Israel, and Nigeria. These countries had negative values except Turkey. A negative net value indicates a weak response; the lower the value, the weaker the response. Egypt is the least COVID-19 responsive. The Scenario 2 results show the lack of response preparedness, in consensus with Eissa [42] who identified pre-COVID-19 gaps in pandemic preparedness, and recommended a neo-mindset in structural public health expenditure—a facilitator of pandemic preparedness.

### Scenario 3 Health Workers’ Status

Table 4 provides rankings based on HCW capacity and minimum COVID-19 infection/ mortality.

**Table 4:** Ranking results of the countries based on the status of health workers.

Scenario 3 HCW Status									
Rank	Country	Net	Phi+	Phi-	Rank	Country	Net	Phi+	Phi-
**1**	South Korea	0.2851	0.3183	0.0332	**12**	Russia	-0.0581	0.1796	0.2376
**2**	New Zealand	0.2531	0.3643	0.1111	**13**	China	-0.0667	0.1716	0.2382
**3**	UAE	0.2222	0.2790	0.0568	**14**	Egypt	-0.0735	0.1738	0.2473
**4**	Qatar	0.2195	0.2783	0.0587	**15**	UK	-0.0790	0.1414	0.2204
**5**	France	0.1387	0.2594	0.1207	**16**	Turkey	-0.0877	0.1317	0.2193
**6**	Germany	0.1182	0.2684	0.1501	**17**	Sudan	-0.1059	0.0804	0.1863
**7**	Saudi Arabia	0.1035	0.2250	0.1215	**18**	USA	-0.1111	0.1417	0.2528
**8**	Israel	0.0930	0.2232	0.1302	**19**	Brazil	-0.1120	0.1344	0.2464
**9**	Italy	0.0245	0.2000	0.1755	**20**	South Africa	-0.1137	0.1285	0.2422
**10**	Nigeria	-0.0148	0.1867	0.2015	**21**	Mexico	-0.2907	0.0530	0.3438
**11**	India	-0.0423	0.1661	0.2085	**22**	Spain	-0.3025	0.0893	0.3918

South Korea has the highest and lowest positive and negative values, the highest HCW capacity with the lowest COVID-19 mortality. New Zealand, UAE, Qatar, France, Germany, Saudi Arabia, Israel, Italy, and Nigeria are in the top ten. All Middle East countries (except Egypt) and notably, only one African country (Nigeria) is ranked in the top ten. However, these countries have a negative net value, indicating a weak HCWs capacity. India, Russia, China, Egypt, UK, Turkey, Sudan, USA, Brazil, South Africa, and Mexico have the lowest net values with Spain ranked the worst. Some of these countries have very high HCWs capacity but high HCWs mortality, and vice versa. For example, Spain has one of the highest number of doctors/1k, but is the worst for HCWs COVID-19 mortality rate. HCWs are the first line of defense against COVID-19 and therefore must be protected to maintain their capacity.

### Scenario 4 COVID-19 Status

Table 5 shows the results for Scenario 4.

**Table 5:** Ranking results of the countries based on COVID-19 status.

Scenario 4 COVID-19 Status/Management									
Rank	Country	Net	Phi+	Phi-	Rank	Country	Net	Phi+	Phi-
**1**	New Zealand	0.2333	0.3242	0.0909	**12**	Germany	0.0283	0.2216	0.1934
**2**	South Korea	0.1927	0.3038	0.1111	**13**	South Africa	-0.0140	0.1950	0.2090
**3**	Qatar	0.1821	0.2931	0.1109	**14**	UK	-0.0427	0.1787	0.2214
**4**	UAE	0.1654	0.2847	0.1193	**15**	Italy	-0.0990	0.1579	0.2570
**5**	Nigeria	0.1184	0.2612	0.1427	**16**	Mexico	-0.1079	0.1459	0.2537
**6**	Sudan	0.1015	0.2527	0.1512	**17**	Russia	-0.1236	0.1549	0.2786
**7**	China	0.0930	0.2633	0.1703	**18**	India	-0.1665	0.1188	0.2853
**8**	Israel	0.0873	0.2457	0.1583	**19**	Brazil	-0.1696	0.1171	0.2868
**9**	Saudi Arabia	0.0644	0.2342	0.1698	**20**	France	-0.1966	0.1078	0.3044
**10**	Egypt	0.0634	0.2391	0.1757	**21**	Spain	-0.2193	0.0923	0.3116
**11**	Turkey	0.0350	0.2195	0.1845	**22**	USA	-0.2255	0.0894	0.3149

New Zealand and South Korea are ranked the most effective in managing COVID-19, minimizing infection rate, and recovery/survival rate. Studies recommend that countries emulate the success of New Zealand and South Korea in managing COVID-19. Accordingly, Qatar, UAE, Nigeria, Sudan, China, Israel, Saudi Arabia, and Egypt are among the top ten. Here, all African and Middle East countries are in the top ten except South Africa. Most importantly, no European country is in the top ten. Turkey is 11^th^ (highest in Europe), then Germany, South Africa, UK, Italy, Mexico, Russia, India, Brazil, France, and Spain, with negative values, indicating bad COVID-19 status. On the downside, USA is the worst in this scenario.

### Full ranking: Holistic capacity response and healthcare resilience

Combining all indicators in all scenarios, an overall ranking of the quantified COVID-19 holistic response capacity and healthcare resilience for each country is shown in Table 6. South Korea obtained the highest net value (0,1945), the most holistic capacity response and resilient healthcare while Mexico is the least, with the lowest net value (-0,1428).

**Table 6:** Full ranking of countries’ healthcare capacity response capacity and resilience.

Full Ranking									
Rank	Country	Net	Phi+	Phi-	Rank	Country	Net	Phi+	Phi-
**1**	South Korea	0.1945	0.2868	0.0923	**12**	France	-0.0012	0.1871	0.1883
**2**	Germany	0.1476	0.2764	0.1288	**13**	Israel	-0.0029	0.1809	0.1838
**3**	China	0.1064	0.2500	0.1436	**14**	Russia	-0.0084	0.1915	0.1999
**4**	New Zealand	0.0974	0.2667	0.1693	**15**	Brazil	-0.0239	0.1839	0.2078
**5**	Saudi Arabia	0.0370	0.1980	0.1609	**16**	South Africa	-0.0399	0.1689	0.2088
**6**	UAE	0.0332	0.1853	0.1520	**17**	India	-0.0793	0.1464	0.2257
**7**	UK	0.0329	0.2004	0.1674	**18**	Spain	-0.0865	0.1568	0.2433
**8**	Qatar	0.0294	0.1845	0.1551	**19**	Egypt	-0.0893	0.1502	0.2395
**9**	Turkey	0.0190	0.1944	0.1754	**20**	Nigeria	-0.1034	0.1404	0.2438
**10**	USA	0.0056	0.1948	0.1892	**21**	Sudan	-0.1301	0.1170	0.2471
**11**	Italy	0.0046	0.1894	0.1847	**22**	Mexico	-0.1428	0.1199	0.2627

The full ranking reveals South Korea’s combined relative high performance in all scenarios, suggesting that it is the most resilient country. South Korea has the highest hospital bed/1k (124.3), extensive health insurance (HI) coverage, and is very transparent, etc. On the other hand, the country has low BMEs/P (0.05%). Other studies have identified the successful mitigation and management of the COVID-19 pandemic in the country. DKG’s [17] COVID-19 treatment efficiency ranked South Korea 3^rd^. You [43] reviewed and concluded that South Korea’s public health policy is a model for other countries. Jackie Oh et al. [44] Identified South Korea’s rapid response improvement within the first 90 days in testing, digital contact tracing, and the first locally made mobile testing centers (later adopted globally). Germany, China, and New Zealand are ranked 2^nd^, 3^rd^, and 4^th^ respectively. Germany, has the highest doctors/1k (4.3), and is among the most innovative, transparent, and independent. DKG [17] ranked Germany 1^st^ for COVID-19 treatment efficiency.

China has the highest ventilators/TC (66.76%), ICU beds/TC (51.03%), vaccine doses (3.6x108), and BMEs/1k (49%). BMEs were identified as unsung or hidden heroes during the COVID-19 pandemic for their active mechanical support and point of care [45, 46]. DKG [17] ranked China 2nd in COVID-19 treatment efficiency. China’s unprecedented quarantine policy helped reduce COVID-19 spread [47]. According to Yin et al. [33], China used experience from the SARS 2003 epidemic, while Cao et al. [48] identified gradual strides in emergency response, monitoring, systematic warning, healthcare reserves restocking, and effective logistics. Ning et al. [49] concluded that China’s success was due to effective public health, providing multi-sectoral synergy capacity with the strategic goal of severing transmission through citizens’ engagement and nationwide awareness. However, China is the highest in TC/P (0.0062%), AC/TC (0.2715%), and the least transparent due to the early cover-up in the country. Furthermore, Cao et al. [48] criticized China for its hesitancy in providing emergency funds, operational efficiency, and decision systems, especially at the early stage. Yin et al. [33] blamed these flaws on previous disparities, which were quickly resolved.

New Zealand has the highest hospital beds/TC (10.84) and extensive HI coverage. It has the lowest ICU beds (176) and ventilators (334); however, the country performed well when the ratio each of these indicators per population and TC is taken into account. New Zealand was ranked in the top three in the COVID-19 model for public health and strategic commitment, examined by Collins et al. [50]. Fouda et al. [5] ranked New Zealand the most effective in its early COVID-19 responses. Similarly, Fernandes [9] identified Germany, New Zealand, South Korea, and China as having high healthcare resilience. Saudi Arabia, UAE, UK, Qatar, Turkey, USA are ranked in the top ten accordingly. USA was most COVID-19 prepared, and a global leader in medicine, innovation, and health technology. It had the highest ventilators/P (0.0483%), Nursing/1k (14.55), and independence in the pandemic. However, USA was the third epicenter, with the highest number of TC, AC, total deaths, and infected HCWs. Salihu et al. [6] found that Germany, Turkey, South Korea, and USA were among the top countries in the Pandemic Efficiency Index (PEI) in terms of the COVID-19 mortality rate. Italy is ranked 11^th^; despite being overwhelmed as the second epicenter, it performed better than half of the countries in this study. Italy was transparent with COVID-19 data, with extensive HI coverage. The robust preparedness and response helped the USA and Italy to mitigate COVID-19, despite being epicenters of the virus.

Furthermore, Saudi Arabia, UAE, and Qatar had high performance due to nationwide testing and other drastic measures against COVID-19. Saudi Arabia has extensive HI coverage, ranked 5th ahead of UAE, Qatar, UK and USA, which concurs with the results from Hale et al.’s [14] OXCGRT. Saudi Arabia’s experience from MERS, as Algaissi et al. [51] noted, provided the country with COVID-19 frontline capacity. The government offered free testing and suspended ≈2.5 million pilgrims for the 2020 Hajj, preventing further catastrophic spread [52]. Similarly, UAE (6th) had the highest ratio of recovery to TC (98.02%) and total tests/P (3.002%), but a low BMEs/1K. Moonesar et al. [16] ranked the UAE above the USA and China; however, China ranks higher than both countries in our study. Qatar is 8th with the lowest mortality rate (0.1579%), consistent with the findings of Omrani et al. [53], extensive HI coverage and a very low HCWs mortality rate. Al Khal et al. [8] identified Qatar’s well-organized, coordinated healthcare services, and public health response to COVID‑19. Because certain public data were publicly unavailable, even after searching in both English and Arabic languages, transparency for Qatar, UAE, and Saudi Arabia was quite low.

Turkey is ranked 9^th^, having implemented the best lockdown and extensive HI coverage. Bakir [54] emphasized the Turkish government’s early and swift implementation of public health measures like physical distancing, travel bans, quarantines, and personal protection. The success of public health and social measures depends on adequate health resources, personnel and financial efforts [55]. France, Israel, Russia, Brazil, South Africa, India, Spain, Egypt, and Nigeria accordingly are in the top 20, with negative net values. France has the highest AC/TC (90.77%) and the lowest TR/TC (6.9129%). DKG [17] ranked France as one of the top five riskiest COVID-19 countries. Israel has the highest ICU beds/P (8.8957%), vaccine doses, and extensive HI coverage. However, Israel has the highest TC/P (8.8957%) and lowest nursing/1 k (33%). DKG [17] ranked Israel above Germany, South Korea, Australia, and China in COVID-19 safety; however, the country is ranked 13^th^ in our study, below these countries. Russia has a low BMEs/1K, but it has the highest testing labs/P (0.0006%), and extensive HI coverage. Brazil has a low BMEs/1K, but provided the highest COVID-19 emergency medical funds. Brazilian COVID-19 researchers had early access to governmental emergency research funds [56]. The Brazilian government provided ≈$9.57 billion in emergency funds across Brazil [57]. However, COVID-19 overwhelmed Brazil’s health system in some regions, resulting in high mortality rates [58].

South Africa has the highest emergency fund/TC, but a low BMEs/1K. India has the highest COVID-19 recoveries, labs, ICU beds, lowest HCWs infection rate (1.1852%) and tied Saudi Arabia for the second highest recovery/TC (97.13%). However, India is lowest for hospital beds/1k (5.3), hospital beds/TC (0.0478%), emergency fund/P, and emergency fund/TC. In early 2021, India became the latest COVID-19 epicenter, as clearly predicted by Kapoor et al. [59], who recommended the mobilization of additional resources to handle severe COVID-19 cases. Spain has the lowest ICU beds/TC (1.4317%), but the highest HCWs mortality and mortality rates (51.29). Spain, Italy, and France have the lowest PEI [6]. Also, DKG [17] ranked Spain among the top 5 COVID-19 riskiest countries. Egypt is ranked 19^th^ with a weak cumulative performance in most indicators. Unfortunately, Egypt has low transparency. Nigeria performed considerably well in TC/P, TR/TC, with unexpectedly high transparency, providing daily COVID-19 updates. Amzat et al. [10] identified the successes of Nigeria in terms of its nationwide lockdown, travel ban, and community mitigation. However, Nigeria has the lowest ICU beds/P (0.0002%), ventilator/P (0.0005%), doctors/1k (0.2), low BME/1k (0.005), and very poor HI coverage. Nigeria’s weak performance is due to the country’s weak preparedness towards pandemics and low physician/patient ratio [60]. Nigeria’s underfunded public hospitals only received t 4% of the annual health budget in 2020, which was insufficient for resource mobilization.

Sudan and Mexico attained the lowest net values, suggesting that they are the least healthcare resilient. Sudan is ranked lowest for HYS score (26.2), test/P (0.3622%), innovations, and vaccine doses/P (0.3198%). Sudan was largely dependent on COVID-19 imports and foreign aid. However, Sudan performed well in TR/TC. Sudan’s weakness was due to its transitional government, fragmented HYS, inadequate tests, hospitals, citizens’ noncompliance, and poor HCWs status [61]. Additional factors included the chronically underfunded health system, dilapidated health infrastructure, lack of HCWs, vital supplies, and poor hygiene practices [62]. Conflicts and poor synergy among key decision-makers led to resignations, accusations, and poor management, further weakening the response. Poverty, ignorance, and persistent economic crisis, according to Sulaiman et al. [63], were major obstructions to Sudan’s response. Sudan, Nigeria, and other sub-Saharan African countries need improvement in critical areas of systematic surveillance, HYS, funding, and research [64]. Most low and lower middle-income countries had limited critical-care capacity to cope with severe COVID-19 cases [11]. Mexico is the weakest performing country in all indicators (lowest ventilators/TC, and highest deaths/TC (8.8767%)). It had the worst case-fatality in Americas [34]. This poor performance relates to the country’s lack of preparedness, resource, and underestimation of COVID-19 risks early in the pandemic [65]. HCWs deaths caused by PPE shortages devastated Mexico, with a deficit of 200k doctors and 300k nurses. Lastly, Mexico suffered from early response hesitancy and distrust between citizens and COVID-19 authorities [65].

## Recommendation and conclusion

In conclusion, COVID-19 is still evolving with new variants, patterns, and epicenters even in 2023; therefore, it is not possible to fully decode the true situation until it is over. Moreover, several countries continue to improve their production, procurement of resources and health workforce, while others are becoming overwhelmed. Perhaps future studies can help identify change in patterns of countries’ capacities, and how they translate to responses. Some countries with fewer resources have managed to keep the number of registered cases relatively low compared to more developed countries. For example, developing countries, especially those in Africa, lack research into COVID-19. However, despite their unpreparedness and weak responses, African countries had lesser infections and mortality compared to some better equipped countries. This situation certainly raises important questions that need to be addressed. Future studies can investigate the efforts and responses of African countries in the fight against COVID-19 and other public health threats capable of causing pandemics. One factor to consider is the demographics and population density in African countries, which may have contributed to the lower number of confirmed COVID-19 cases. In addition, a variety of factors, including climate, diet, physical fitness, and cultural traditions, may have influenced the differences in the number of confirmed cases across countries. Further future research and analysis is needed to fully understand the reasons for these differences and to identify further preparedness measures.

Our results ranked the COVID-19 responses of 22 countries, considering the dynamics of COVID-19. The study is sensitive to weighting, additional parameters, and the ever-changing dynamics of COVID-19. The implications of using outdated data was emphasized, which may have affected effective decision-making and implementation of measures. Governments and international organizations should improve transparency for a clearer depiction. With updated and transparent data, future studies can implement Fuzzy-PROMETHEE or other MCDM techniques to clearly evaluate countries’ responses to COVID-19. Lastly, crises are opportunities to question the reality of entire societies to reinvent and evolve toward an improved, organized, inclusive, responsive, and resilient system. Our intention is for this study to contribute to discussions and actions to improve global health systems.
